# Tick infestation risk for dogs in a peri-urban park

**DOI:** 10.1186/1756-3305-6-358

**Published:** 2013-12-17

**Authors:** Amy L Jennett, Faith D Smith, Richard Wall

**Affiliations:** 1 Veterinary Parasitology and Ecology Group, School of Biological Sciences, University of Bristol, BS8 1UG Bristol, UK

**Keywords:** Climate, Dogs, Habitat, Management, Tick-borne disease, Tick, Vector, Urban, Zoonosis

## Abstract

**Background:**

Increases in the abundance and distribution of ticks and tick borne disease (TBD) within Europe have been reported extensively over the last 10–20 years. Changes in climate, habitat management, economic patterns and changes in the abundance of hosts, particularly deer, may all have influenced this change to varying extents. Increasing abundances of tick populations in urban and peri-urban environments, such as parks, are of particular concern. In these sites, suitable habitat, wildlife hosts, tick populations, people and their pets may be brought into close proximity and hence may provide foci for tick infestation and, ultimately, disease transmission.

**Methods:**

The distribution and abundance of ticks were examined in an intensively used, peri-urban park. First the seasonal and spatial distribution and abundance of ticks in various habitat types were quantified by blanket dragging. Then the pattern of pet dog movement in the park was mapped by attaching GPS recorders to the collars of dogs brought to the park for exercise, allowing their walking routes to be tracked. Information about the dog, its park use and its history of tick attachment were obtained from the dog-owners.

**Results:**

Ticks were found predominantly in woodland, woodland edge and deer park areas and were least abundant in mown grassland. Tick infestation of dogs was a relatively frequent occurrence with, on average, one case of tick attachment reported per year for a dog walked once per week, but for some dogs walked daily, infestation 4–5 times per week was reported. All dogs appeared to be at equal risk, regardless of walk route or duration and infestation was primarily influenced by the frequency of exposure.

**Conclusions:**

In peri-urban green spaces, tick-biting risk for dogs may be high and here was shown to be related primarily to exposure frequency. While tick-biting is of direct veterinary importance for dogs, dogs also represent useful sentinels for human tick-exposure.

## Background

Ticks are important vectors of pathogens to humans, domestic animals and wildlife worldwide. The distribution and abundance of a range of tick species appear to have increased in recent years and these changes have been attributed variously to changes in factors such as habitat management, climate, economic patterns, travel and changes in host abundance, particularly an increase in the number of wild hosts such as deer [1-5]. Each of these factors may carry a different weight and play a different role under specific local circumstances.

The most widespread tick of medico-veterinary concern in north-western Europe is *Ixodes ricinus*, which is the known principal vector for the agents of Lyme borreliosis and tick borne encephalitis (TBE) in humans and *Babesia*, *Anaplasma*, louping ill virus (LIV) and various strains of *Rickettsia* in domestic and companion animals [6-8]. Studies of the ecology and pathogen transmission by this tick species have most frequently focussed on rural habitats; *I. ricinus* is most commonly associated with woodland and moorland where the density of wild hosts is high [9]. Its distribution is largely determined by two factors: microclimate and the availability of hosts, which are in turn both strongly associated with vegetation type [10]. Woodland and transition zones, where two different vegetation types meet, are regarded as primary habitats for these ticks largely due to the fact that such areas attract a greater variety and abundance of hosts [11-13]. The thickness and quality of the mat layer (the layer of decaying vegetation lying next to the soil) appears to be of particular importance to provide ticks with the necessary humidity to prevent desiccation [14]. However, high tick densities and a high prevalence of infection have also been found associated with urban recreational spaces [15]. For example, in Helsinki, 32% of *I. ricinus* sampled were found to be infected with *Borrelia burgdorferi*[16]. In western Germany the risk of infection from Lyme disease within an urban environment was found to be very similar to the risk associated with rural woodland [17]. In such urban environments the limited number of green spaces available are, of necessity, used by wildlife as habitat for survival and by people and their companion animals for recreation. While suitable habitat for large hosts may be lacking in these environments, rates of disease transmission may be exacerbated by the constrained proximity of smaller mammalian wild hosts, humans and their companion animals, particularly dogs [18]. It is important therefore to develop an understanding of interactions between vectors and hosts in these specific habitats so that effective advice can be offered about how best they may be managed so that disease risk is minimised. Hence, the aim of the present study was to investigate the interaction between ticks and the domestic dogs being brought for exercise to a large peri-urban park in south west England.

## Methods

### Study site

**Figure 1:**
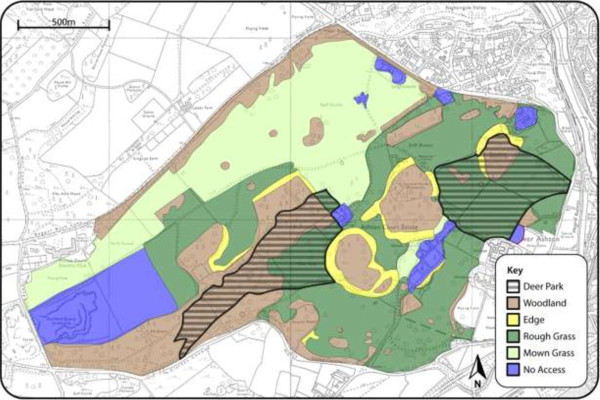
**Seasonal pattern of abundance of *Ixodes *** **ticks as indicated by the proportion of 10 m long blanket drags (1 m**^**2**^**) that collected more than one larva, nymph or adult at Ashton Court.**

The study site was the Ashton Court Estate (51.4479° N, 2.6446° W). It is a recreational park located in south west England, immediately to the west of the city of Bristol. The site ranges between 10 m and 120 m above sea level, is distributed over 500 hectares and includes areas of deciduous woodland, rough grassland, ponds and a managed grassland golf course (Figure 1). There are two enclosed deer parks, one to the east containing red deer (*Cervus elaphus*) and to the west, fallow deer (*Dama dama*). Wild roe deer (*Capreolus capreolus*) roam freely across the entire site. Public access to the red deer enclosure is permitted, as long as dogs remain on leads, but there is no public access to the Fallow deer enclosure. The wildlife present, in addition to the deer, includes wild rabbits, rodents, foxes and birds and it is occasionally grazed by cattle and sheep as part of its routine management. Ashton Court is heavily used for recreational activities including: cycling, golf, horse riding, orienteering, dog walking and annual festivals. In 2007 it recorded approximately 1.7 million visitors, making it the most intensively used park in south west England. This study was undertaken with the permission of the University of Bristol ethical approval committee.

### Tick sampling

High resolution maps (10×10 m) of Ashton Court were produced. Each 10 × 10 m square was assigned to a habitat type category, according to the dominant vegetation type that covered most of its surface area. This was determined by visiting each site, ground surveying and matching the ground surveys to aerial photographs of the sites. Habitat type was divided into five categories following the Land Cover Map 2000 [19]: grassland, woodland, edge habitat, unmanaged grassland with an enclosed population of deer and unmanaged grassland without an enclosed population of deer. The perimeter of each habitat type was then marked on the map manually using a hand held GPS (GARMIN, GPS map 76CSX). GPS coordinates, marking the perimeter of each habitat type were uploaded onto an Ordinance Survey (OS) map in ArcMAP v9.0 and converted to shape-files (Figure 1).

The entire site was sampled for ticks twice a week from March to October in 2010. Prior to each sampling occasion, five sample locations in each habitat type were chosen using a random number generator. The random numbers were associated with map coordinates. Once at the site, a portable GPS was used to locate these pre-selected sites. At each sample location, standardised 10 m blanket drags were conducted to measure levels of tick activity, following the protocol of Milne [20]. The dragging apparatus consisted of a white coloured cotton sheet (1 × 1 m), held square with a 125 cm long wooden pole of 1 cm diameter. The 10 m drag began at the location of the randomly generated coordinate and the length was marked out with a measuring tape [21]. Hand-held data loggers were used to record the ground and air temperature, humidity, wind-speed and vegetation mat depth at each drag location.

All adult and nymphal ticks found attached to the cloth after a drag were counted and removed from the cloth at the site and placed in a labelled tube for later identification in the laboratory [22]. Because the aim of this part of the study was simply to obtain a representative estimate of the relative distribution and seasonal abundance of ticks in the park and, given the difficulty inherent in their identification, larval ticks were simply counted, removed from the cloth and returned to the vegetation.

### Dog recruitment

From October 2012 to December 2012 and in April 2013, people entering Ashton Court with their dogs for regular exercise were approached and asked to complete a short questionnaire to gain information about the age, breed, sex and neutered status of their dog. Other questions were included to obtain information about the frequency with which they walked in Ashton Court, their history of tick infestation, ectoparasite treatment and their ability to identify a tick. For the latter, owners were asked to identify a photograph of a fully engorged adult female tick (*I. ricinus*) from among pictures of a house fly (*Musca domestica),* cat flea (*Ctenocephalides felis*) and a sucking dog louse (*Linognathus setosus*).

The only selection requirements for initial inclusion in the study were that the owners walked their dog in Ashton Court at least once per month and that they would give consent for their dogs to be tracked via a GPS (Global Positioning System). Owners gave their answers verbally and the investigator entered the data on the questionnaire. On obtaining consent to track their dogs via GPS and owner completion of the questionnaire a GPS tracker (I-gotU GT 100, Mobile Action Technology Inc., Taiwan) was attached to the collar of the participating dog. The owner and their dog were then allowed to go on their routine walking route. The trackers were collected on the return of the owner and their dog at the end of their walk. A maximum of five dogs could be tracked at any one time; if any dog walker had multiple dogs, only one dog from the group was tracked and this one was chosen at random. To avoid bias, since many dog walkers reported that they walked a similar routine route on each visit to the park, owners were only included in the study once, and on subsequent days were excluded from selection.

### Analysing dog walk routes

Data files were downloaded from the tracker onto a computer and imported into ArcMAP (v9.0). The total length of each walk was then calculated and the exact routes overlaid onto the OS map within ArcMAP. GPS coordinates of each walk were joined chronologically and converted into shape-files, enabling each route to be viewed individually and compared with the known habitat type. The distance each dog spent in each habitat type was measured and recorded. All walks recorded could be assigned to one of five walk-type categories (Table 1). Walk type was assessed by examining the percentage of the total walk a dog spent within each habitat type, based on the patterns of habitat use observed.

**Table 1:** The classification of five walk types undertaken by dogs that were tracked on their visit to Ashton Court Estate

**Walk type**	**Classification**
1	100% of walk spent in grassland (mown and rough).
2	The majority of walk spent in grassland (mown and rough) with a minority (≤30%) spent in woodland habitat.
3	Walk length was evenly split (50/50) between woodland habitat and grassland habitat (mown and rough).
4	The majority of walk was spent within the deer parks.
5	Walk distance split evenly across all habitat types.

### Statistical analysis

The primary dependent variable used in the analysis was infestation frequency. This was based on the number of occasions on which owners stated that they usually found ticks on their dog each week over the previous year. Since this was likely to be dependent on the number of times a dog walked in the park, for statistical analysis, infestation frequency was expressed as the number of occasions on which ticks were found on a dog per week, per walk. Differences in infestation frequency associated with sex, breed, hair length (long, medium short), temperament (on an arbitrary scale of 1 to 10, depending on the owner’s perception of dog independence), ectoparasite treatment and walk type (Table 1) were analysed using chi-squared tests. Possible associations between tick infestation frequency and total walk length, walk frequency (number of visits per week) and dog age (years) were analysed using multiple regression (Statgraphics V.16.1, Statpoint Technologies Inc). The distance walked by each dog was then further sub-divided into the individual distances spent in each separate habitat type and possible associations with infestation frequency analysed using regression.

## Results

### Seasonal tick abundance and habitat distribution

Between March 1^st^ and October 31^st^ 2010, 1,500 blanket-drags were undertaken. A total of 728 larvae, 584 nymphs and 47 adults were collected; 17.3% of drags recorded at least one adult or nymph. All nymphs and adults collected were identified as *I. ricinus*; since the larvae were not identified to species they will be referred to only as *Ixodes* spp.

The pattern of tick collections in the blanket drags was largely bimodal: on the majority of occasions no ticks were collected, where ticks were found, the modal classes were 1 or 2 ticks per drag only. Hence, mean tick count numbers were considered to be a relatively uninformative metric and the percentage of blanket drags that collected one or more tick was used as the measure of tick presence. The percentage of blanket drags that collected larvae was relatively low compared to numbers of adults and nymphs (7.2%), but when they were present, high numbers were observed (>130), indicating a highly aggregated population distribution.

**Figure 2:**
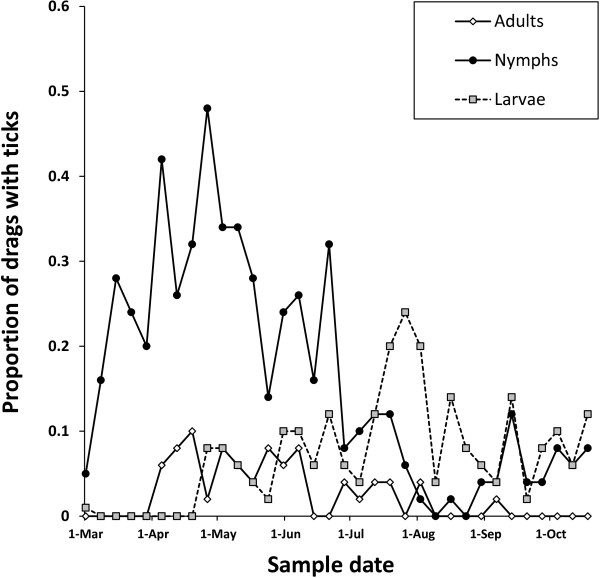
**Seasonal pattern of abundance of *Ixodes *ticks as indicated by the proportion of 10 m long blanket drags (1 m**^**2**^**) that collected more than one larva, nymph or adult at Ashton Court.** All nymphs and adults were identified as *Ixodes ricinus* and larvae only as *Ixodes* spp.

Larvae did not become active until mid-April, activity was then relatively low until July after which the number of drags which collected larvae peaked (Figure 2). Larvae continued to be found by the blanket drags throughout autumn. The activity of *I. ricinus* nymphs rose steadily throughout the spring to peak in early May. The numbers of drags that collected ticks continued to be high until the end of June, fell to almost zero in midsummer and then showed a slight small rise in September. Adults were the least abundant in the drags at all times of year. Adults were first collected at the beginning of April and this continued throughout the spring. Low levels of adult activity were observed during the summer months and almost no adult ticks were observed questing in the autumn (Figure 2).

**Figure 3:**
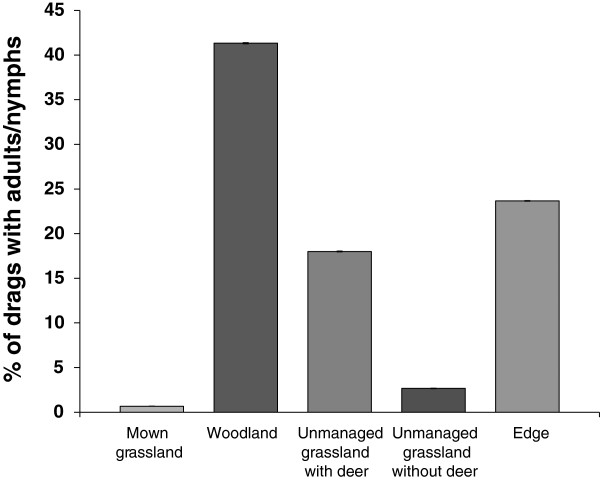
**Percentage (± 95% CI) of drags with adult or nymphal *Ixodes ricinus *** **ticks in each different habitat type surveyed.**

There was a significant difference in the percentage of drags that collected ticks in the various habitats at Ashton Court (χ^2^ = 232.9, *d.f*. = 1,495, *P* < 0.001). Most ticks were found in the woodland habitat, with relatively high numbers of drags with ticks found in the edge and unmanaged grassland with deer habitat. Conversely, very few drags collected ticks in the unmanaged grassland without deer, and ticks were only found on two drags in the grass habitat (Figure 3). The presence of ticks was significantly associated with a lower mean saturation deficit (t = −2.1, *d.f.* = 415, *P* = 0.03) and a greater mat depth (t = 2.87, *d.f*. = 393, *P* = 0.004).

### Dog study population

A total of 125 dog walkers who visited the park more than once a month were recruited to the study; 86 between October and December 2012 and 39 in April 2013. There were no differences in the results obtained at these two time periods, so the data are combined in all further analysis.

Of the dogs sampled, breed class varied widely, with gundog, mixed and terrier breed categories dominating (30.4%, 22.4% and 18.4% respectively). All other individual breed categories each composed <10% of the sample size. Neutered dogs accounted for 64% of the sample; of these, half were neutered males and half neutered females. The remaining un-neutered population was divided into 42% entire male and 58% entire female. Age distribution and temperament scores (likelihood to stray away from owner and explore the park) were normally distributed. The average distance walked by dogs at each visit was 4.2 km (SD ± 1.85 km).

All owners responded “yes”, when asked if they knew what a tick looked like. However, 11% (14) of the owners were unable to identify the tick correctly from the four photographs that they were shown. Of these, 12 were owners who reported that their dog had never had a tick. The owners who correctly identified a tick, were found to be more likely to report higher frequencies of tick infestation than the owners who could not. In all subsequent analyses, therefore, the results collected from the dogs of owners who were unable to correctly identify a tick have been disregarded in subsequent analysis, since their responses were considered to be unreliable, leaving a final sample for analysis of 111 recorded dog walks.

### Tick infestation frequency

**Figure 4:**
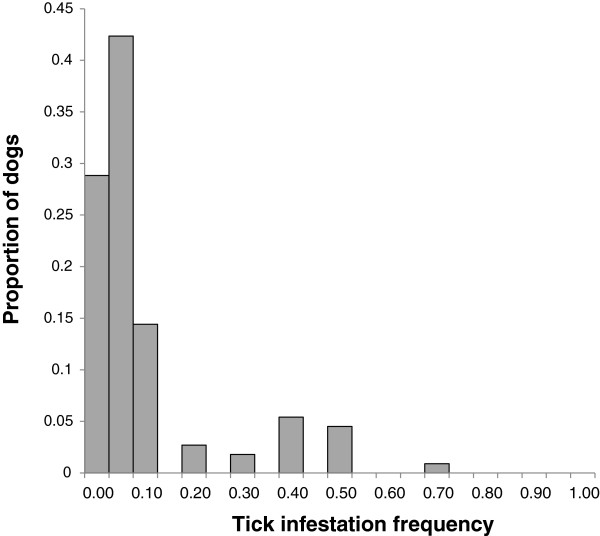
**The proportion of dogs that reported different frequencies of tick infestations per visit per week.** The number of tick infestations per visit was calculated from the reported numbers of tick infestations and the reported walk frequency for dogs walked in Ashton Court. N = 111 (excluding owners who were unable to identify a tick).

Amongst owners, 87% identified spring and early summer as the time when they were most likely to see ticks on their dogs and 72% thought that the woodland was the habitat from which their dogs were most likely to acquire ticks. In most cases (96%) the owners only reported the presence of one tick on each infestation occasion. The distribution of tick infestation frequency was significantly more overdispersed than the Poisson distribution that would have been expected if tick attachment was a purely random event (χ^2^ = 1001.7, *d.f.* = 11, *P* < 0.001, Figure 4). The median frequency of infestations was 0.02 per walk/week.

For further analysis, dogs were categorised into tick infestation frequency groups: never (no tick infestation), low (less than 0.03), medium (0.04-0.1) and high (>0.2) cases. 29% of owners reported that they never had ticks, 31% reported low infestation frequencies, 25% medium infestation frequencies and 15% reported high frequencies of tick infestation. There was no difference in tick infestation frequency between male, female, neutered males or neutered females (χ^2^ = 12.8, *d.f.* = 9, *P* = 0.17). There was no difference in tick infestation frequency between dogs that had been recently treated with acaricide or dogs that had not (χ^2^ = 6.96, *d.f.* = 6, *P* = 0.32). There was no difference in the frequency of reported tick infestations between dog breed groups (χ^2^ = 4.04, *d.f.* = 9, *P* = 0.91). There was no difference in the frequency of tick infestations of dogs with a temperament score of below or above 5 (χ^2^ = 3.06, *d.f.* = 3, *P* = 0.38). Short-haired dogs were significantly more likely to be in the highest infestation category than long-haired dogs (χ^2^ = 17.73, *d.f.* = 3, *P* < 0.001).

There was no relationship between distance walked and the frequency of tick infestation *(*F = 0.18*, P* = 0.67). Most dogs spent the largest fraction of their walk in grassland habitat (80% of dogs) and 18% of dogs walked only in the grassland. The number of dogs that followed walk type 4 (deer park) was too low for statistical analysis. Tick infestation frequency did not vary significantly between the remaining four walk types (χ^2^ = 4.17, *d.f.* = 9, *P =* 0.89). There were no relationships between the distance a dog walked in any particular habitat type and tick infestation frequency *(*F = 0.36, *P* = 0.87).

**Figure 5:**
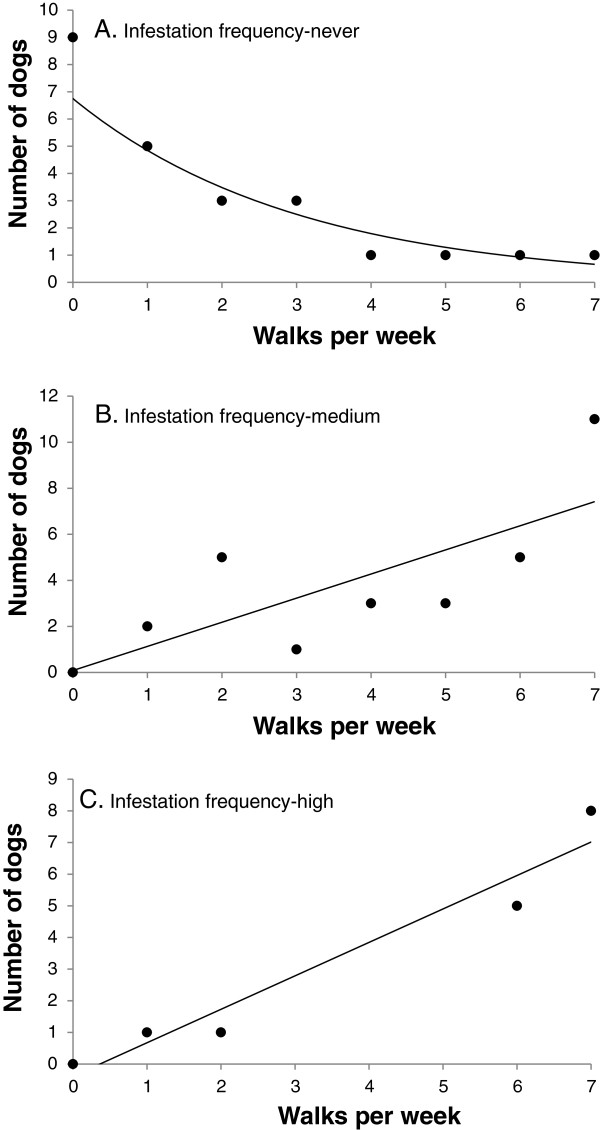
**The number of dogs in different tick infestation categories, that were walked different numbers of times per week.** A: owners who reported that they never saw ticks, Y = exp(1.91 - 0.33*X), F = 38.47, *P* < 0.001, r^2^ = 86.5, n = 24). B: owners reporting a medium infestation frequency, Y = 0.083 + 1.047*X, F = 7.81, *P* = 0.03, r^2^ = 55.7, n = 30). C: owners reporting a high infestation frequency, Y = −0.381 + 1.056*X, F = 45.6, *P =* 0.006, r^2^ = 94.1, n = 15). Data points at 0 represent dogs walked less than once per week but greater than once per month.

Dogs that never acquired ticks from the park walked a median of once per week. In this group, dogs that walked more frequently were significantly less likely to report that they never acquired ticks (Figure 5a). In the low infestation frequency group, there was no relationship between the number of owners reporting ticks and the number of times they walked their dogs. For the medium and high infestation frequency groups, dogs were walked a median of five times per week and the more frequently dogs were walked the greater the number of owners reporting tick infestation (Figure 5b,c).

## Discussion

The examination of 3,534 dogs for ticks, selected at random over a 9 month period at veterinary surgeries throughout the UK, was reported by Smith *et al*. [23]. They found that 810 dogs were carrying at least one tick*. Ixodes ricinus* was identified in 72.1% cases, *Ixodes hexagonus* in 21.7% and *Ixodes canisuga* in 5.6% cases. The incidence of tick attachment was estimated to be 0.013 per day in March (lowest) and 0.096 per day in June (highest). Although the current study used a very different approach to that of Smith et al. [23], the estimated tick infestation rates are not dissimilar. In the present study, the number of reported tick infestations per visit per week ranged from zero to 0.7 with a median frequency of 0.02. For some dogs walked daily, infestation 4–5 times per week at peak tick-biting activity periods was reported. Based on the current data, it can be predicted that, on average, a dog that was walked in Ashton Court once per week might expect to acquire one tick infestation per year, whereas a daily walker might be expected to acquire an average of 7 tick infestations per year. However, in these results, infestation frequency is an estimated annual average around which there will certainly be substantial seasonal variation, since there is a pronounced seasonal change in tick questing activity, with most occurring in spring and early summer and a secondary smaller period of activity in autumn [24]. Unfortunately, the seasonal changes in infestation rate could not be obtained from the questionnaire data collected here because owners were not asked to specify different tick detection rates over time, as it was considered that this would be too difficult for owners to recall with any meaningful accuracy. In future studies, rather than relying on owner detection and recall for estimates of tick attachment, it might be possible to undertake clinical examinations of dogs as they exit the park; however any ticks acquired during a walk may not yet have attached and would be very difficult to detect. Hence, regular follow-up visits to owners, continued throughout a seasonal cycle, might be necessary to obtain more reliable estimates of true attachment rates. Nevertheless, the results presented here do give a useful indication of differences in attachment at a relatively broad level.

To try to increase confidence in data, only those owners able to identify a fully engorged female adult tick were included in the analysis. It is possible that this approach was limited by the fact that the photograph did not accurately depict what the owner might see if a tick was attached to a dog, since the gnathosoma would be hidden. Nevertheless, it is considered that this question was of value and gave some useful indication of the likely lack of recognition among dog owners. Despite the inclusion only of owners who could correctly identify the tick from the photograph shown, it is still likely that tick infestations were under reported. Owners of short haired breeds reported a higher incidence of ticks per visit. Since the detection of ticks depended on owners, with their differing degrees of grooming and detection proficiency, the significantly higher reported tick infestation by the owners of short-haired dogs suggests that either long hair has a barrier effect, protecting these dogs against tick infestation or, more likely, short hair allows owners to notice ticks more readily. The implication of this conclusion is that tick infestation may be detected less frequently in medium and longer-haired dogs.

Here, as expected, in the area of unmanaged grassland where deer were enclosed, the likelihood of finding active *I. ricinus* ticks was 7.43 times higher than in unmanaged grassland without enclosed deer [25,26]. Ticks were rarely found in the unmanaged grassland habitat without enclosed deer, despite wild deer being observed occasionally in this habitat, suggesting the enclosed populations of deer are responsible for creating a localised area of high tick abundance. Although the tick distribution analysis was undertaken in 2010 and the dog infestation analysis in 2012/13, it is not considered likely that the tick distribution would have changed significantly, as the habitat and landscape management in the park remained unaltered over this period. Hence, given the strong associations between habitat type and tick abundance it was expected that dogs that walked longer distances overall and those that walked longer distances in the woodland and woodland edge habitats, would report a higher incidence of tick attachment. Indeed, in North America, current advice on tick bite prevention suggests the avoidance of high risk habitats [27]. It was of interest therefore that here no statistical associations were found between tick infestation frequency and total walk length or the distance dogs spent in the expected high tick risk habitats, such as the woodland or woodland edge. All owners were asked to follow their routine walk when approached to participate in the study. It was therefore assumed that the GPS recordings were accurate representations of their average walk. This finding suggests that the route taken by a dog, and the length of the walk, did not significantly change the risk of tick infestation. This result is most likely to be due to the fact that most dogs walked in a range of different habitat types (79% of dogs spent at least some time in woodland or woodland edge habitat), resulting in insufficient variation in the length or type of walk undertaken by dogs visiting the park to allow significant levels of association with infestation frequency to be detected.

In this study, the number of times that dogs visited Ashton Court was the most significant predictor of tick infestation. This is a similar finding to that of Sonenshine [28], who found that frequency of contact with infested environments influenced the probability of tick attachment and the severity of infestation. Here, owners reporting that their dogs never got ticks walked fewer times per week, with the maximum reported dog walking frequency being less than once per week. Conversely, extremely high infestation only occurred at high walk frequency, with 85% of owners reporting the highest infestation rates walking ≥6 times a week. However, within these general significant trends there was nevertheless some variation. For example some owners walking ≥5 times per week still reported low tick attachment incidence. This variability may be associated with the compounded effects of walk type, distance, hair length and dog breed (which were individually non-significant predictors of risk) it may also include variation in the day-to-day, weather-related questing activity and the precise days walked. Climatic conditions and weather patterns are known to influence questing tick activity [29-32]. In particular, a minimum temperature of approximately 7°C for adult and nymphal life-cycle stages and relative humidities of greater than 80% are generally considered necessary for optimal questing activity [24,30,33,34]. Complex interactions between different factors not included in the questionnaire, such as individual grooming regimes, time of day dogs are walked and whether dogs are walked in all weather conditions or just when conditions are favourable, may also add further to the variation seen in the data.

## Conclusions

The central findings of the present study are that tick infestation of dogs in a peri-urban park is a relatively frequent occurrence and the frequencies reported here are likely to be conservative annual estimates with a considerable degree of under-reporting. All dogs that are walked regularly in this park are likely to acquire at least one tick infestation per year. All dogs appear to be at equal risk regardless of walk route, although owners of short-haired dogs were more likely to report ticks; infestation was primarily influenced by the frequency of exposure in the park. Notably, while tick-biting is of direct veterinary importance for dogs, dogs also represent useful sentinels for human tick-exposure [35]. Avoidance of the deer enclosures is likely to be practical, but avoidance of the woodland and woodland edge used by wild deer, is likely to be impractical (and perhaps undesirable) for most dog walkers. Better veterinary advice to the dog-owning public on tick prevention, detection and removal might therefore be valuable approach to mitigation.

## Competing interests

The authors declare that they have no competing interests.

## Authors’ contributions

ALJ undertook the dog walk survey, its analysis and interpretation; FDS undertook the tick distribution survey. RW devised and supervised the work, contributed to the analysis and interpretation of the data. All authors contributed to the preparation and approved the final version of the manuscript.
